# Supplementary figures and images for: Genetic Diversity, Population Structure and Selection Signature in Begait Goats Revealed by Whole-Genome Sequencing
Source: Animals (Basel). 2024 Jan 18;14(2):307. doi: 10.3390/ani14020307 (PMC10812714; doi:10.3390/ani14020307)

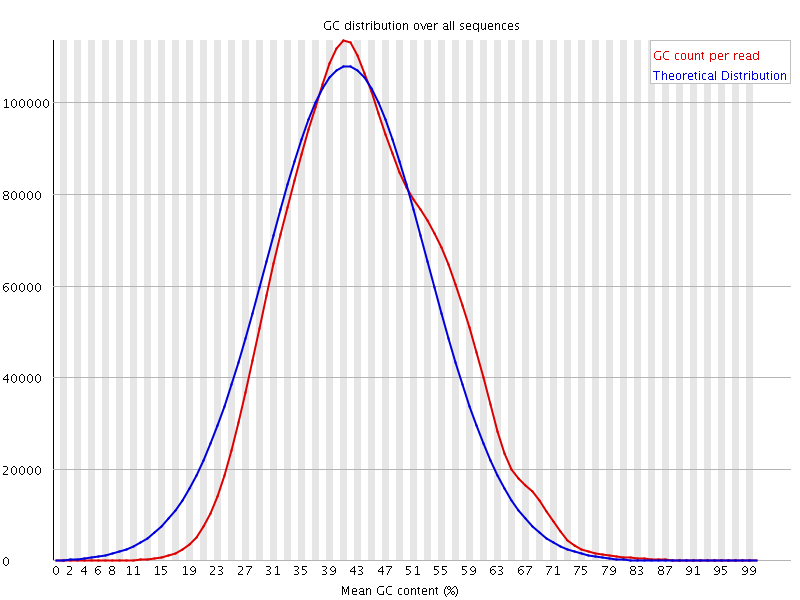

Supplement: Supplementary file 1 [file animals-14-00307-s001.zip › Figure S1.png]
